# Factors associated with an increased risk of osteochondral injuries after patellar dislocations: a systematic review

**DOI:** 10.1186/s13018-023-04265-8

**Published:** 2023-11-01

**Authors:** Zhi Yi, Xiaohui Zhang, Meng Wu, Jin Jiang, Yayi Xia

**Affiliations:** Department of Orthopaedics, Orthopedic Clinical Medical Research Center of Gansu Province, Intelligent Orthopedic Industry Technology Center of Gansu Province, Lanzhou University Second Hospital, No. 82 Cuiyingmen, Chengguan District Lanzhou, Gansu People’s Republic of China

**Keywords:** Osteochondral injuries, Patellar dislocations, Risk factors

## Abstract

**Purpose:**

The purpose of the study was to summarize the available evidence and identify risk factors for osteochondral injuries (OCIs) after patellar dislocations.

**Methods:**

A systematic literature review was conducted in PubMed, Embase, Web of Science, Cochrane Library, and China national knowledge infrastructure from inception to December 22, 2022, according to the preferred reporting items for systematic reviews and meta-analyses guidelines. Studies regarding risk factors for OCIs after patellar dislocations were included. Literature search, data extraction, and quality assessment were performed independently by two authors.

**Results:**

A total of 16 studies with 1945 patients were included. The risk factors for OCIs after patellar dislocation were categorized into four main categories, including demographic characteristics, patellar depth and position, femoral trochlear morphology, and other risk factors in this study. Five and three studies supported the idea that male sex and skeletal maturation may be risk factors, respectively. Normal femoral trochlea (two studies) and complete medial patellofemoral ligament (MPFL) injuries (two studies) may be associated with the development of OCIs. Three studies show that ligamentous laxity or joint hypermobility may prevent OCIs. Patellar depth and position (eight studies) may not be associated with the development of OCIs.

**Conclusions:**

Based on the available evidence, an increased risk of OCIs following patellar dislocation may be associated with male sex and skeletal maturation. Furthermore, normal femoral trochlea and complete MPFL injuries may increase the risk of OCIs, while factors such as ligamentous laxity or joint hypermobility may reduce the risk.

***Level of Evidence*:**

Level IV, systematic review of Level II and IV studies.

## Introduction

Chondral or osteochondral injuries (OCIs) following patellar dislocation are common in active young patients, and these defects impact both short- and long-term outcomes [1–3]. Specifically, these concomitant injuries not only affect the normal function of the knee and cause intense discomfort to the patient, but if they involve damage to the cartilage, synovial membrane, meniscus, subchondral bone, and infrapatellar fat pad, they can further lead to the development of osteoarthritis [1, 4–6]. Although these injuries could cause severe sequelae in affected patients, they are often challenging to diagnose adequately with imaging alone [7–9]. Therefore, knowledge about risk factors for chondral injuries or OCIs would be clinically essential to improve the accuracy of preoperative diagnosis and better surgical planning.

Furthermore, although the literature on the epidemiology of patellar dislocation and its risk factors is extensive [10–16], only a few studies have focused on the risk factors for chondral injuries or OCIs after patellar dislocation and no consistent conclusions have been drawn [2, 17–19].

Given the controversial findings and the lack of studies comprehensively reviewing risk factors for chondral injuries or OCIs, this systematic review aimed to summarize the available evidence and identify risk factors for chondral injuries or OCIs in patients with patellar dislocation. In general, early prevention and appropriate interventions for risk factors of specific injuries are beneficial in improving patient clinical and functional outcomes. We hypothesized that the risk factors associated with cartilage injuries or OCIs include demographic characteristics and anatomical correlates.

## Methods

The findings of this systematic review were carried out following the Preferred Reporting Items for Systematic Reviews and Meta-Analyses (PRISMA) guidelines [20]. Furthermore, the registered number at the International Prospective Register of Systematic Reviews (PROSPERO) is CRD42022349964.

### Literature search and strategy

A systematic electronic search of PubMed, Embase, Web of Science, Cochrane Library, and China National Knowledge Infrastructure Database (CNKI, Chinese database) was performed from inception to December 22, 2022. We performed a search strategy combining Medical Subject Headings (MeSH) terms and free words. We used Boolean logic to connect "patellar dislocation," "patellar instability," "osteochondral," "cartilage," "chondral," "injury," "lesion," "damage," and "fracture." Meanwhile, manual retrievals of the reference lists of the identified articles were conducted for further relevant literature.

### Eligibility criteria

The inclusion criteria were: (1) patients with patellar dislocations; (2) observational studies involving risk factors for chondral injuries or OCIs. The exclusion criteria were as follows: (1) other injuries resulting in OCIs (such as anterior cruciate ligament injuries); (2) studies with duplicate published data; (3) systematic review, animal studies, editorial commentary, and meeting papers.

### Study selection

All obtained articles were imported into Endnote (Clarivate Analytics, Philadelphia, PA, USA) for de-duplication and further management of the remaining articles. Initially, the titles and abstracts identified by the electronic search were independently reviewed by two reviewers (ZY and XZ) to select potentially relevant studies; the full text was later read to determine the final inclusion results. In cases of disagreement between the two reviewers, the third author of this review (MW) was consulted.

### Quality assessment and level of evidence

Quality assessment and grading of the level of evidence were performed independently by two reviewers (ZY and XZ). Methodological quality was assessed using the Methodological Index for Non-Randomized Studies (MINORS) [21]. Since the scores were an ordinal variable, we used the intra-class correlation coefficient (ICC) to assess the agreement between the two reviewers. ICC > 0.90 indicates excellent, 0.75–0.90 indicates good, 0.50–0.75 indicates moderate, and < 0.50 indicates poor [22]. Any disagreements were resolved by the author team discussing them.

### Data collection

Two researchers (ZY and MW) extracted the following information using a predesigned spreadsheet: first author, publication date, country, study design, sample size, demographic characteristics (such as age and gender), diagnosis method, the prevalence of injuries, and associated risk factors. We analyzed patellar depth and position and their associated parameters based on the extracted information. The patellar height was assessed by using the Caton-Deschamps index (CDI), Insall-Salvati index (ISI), and patellotrochlear index (PTI); parameters regarding patellar position also include the lateral patellar inclination (LPI), lateral patellar displacement (LPD), lateral patellofemoral angle (LPFA), and patellofemoral congruence angle (PFCA) [23–32]. The metrics for assessing femoral trochlear morphology include trochlear depth, sulcus angle, lateral trochlear inclination angle (LTIA), trochlear facet asymmetry ratio (TFAR), and trochlear condyle asymmetry ratio (TCAR) [29, 33–35]. Also, the cartilage or osteochondral injuries involved in this study were accessed according to the Outerbridge classification, respectively [36, 37].

### Data synthesis

An emphasis needed to be placed on the fact that OCIs were exclusively defined in this study based on previous studies and the characteristics of the included studies, including cartilage injuries, deep abnormalities of cortical defects, and osteochondral fractures [38, 39]. Studies were heterogeneous regarding participants, methods, and outcomes. Therefore, a meta-analysis was not applicable, and a narrative synthesis was performed. Study findings were grouped and synthesized according to demographic characteristics, patellar depth and position, femoral trochlear morphology, and additional risk factors.

## Results

### Study selection

**Fig. 1:**
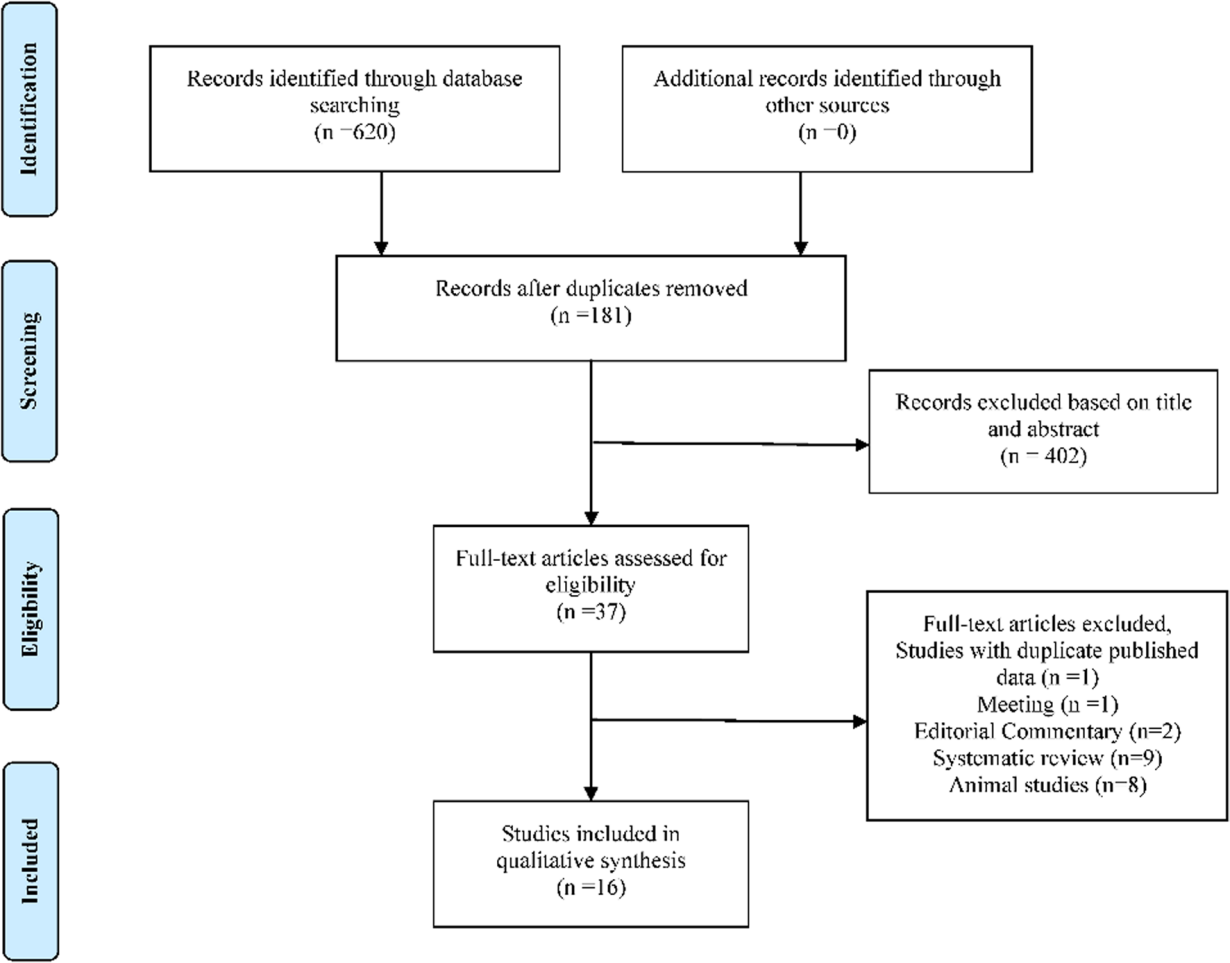
Flow chart of the searching and screening of literature

A total of 620 literature were yielded, and 181 duplicate references were excluded. The remaining 439 studies were reviewed according to titles and abstracts, and 402 articles that did not meet the inclusion criteria were excluded. After reading the full article (n = 37), 16 studies were available for this study (Fig. 1) [2, 17–19, 29, 38, 40–49].

### Characteristics of the included studies

Detailed baseline characteristics of each study were shown in Table 1. A total of 1945 patients with 2002 knees from 16 studies were enrolled. The overall prevalence of OCIs was 57.4%. All included studies were observational, including four prospective cohort studies [19, 45, 47, 49] and 12 retrospective cohort studies [2, 17, 18, 29, 38, 40–44, 46, 48]. For the diagnosis methods of OCIs, 10 studies were based on MRI, five on arthroscopy or surgery, and one on ultrasonography. Most of the included studies were Level 3 evidence (68.75%), followed by Level 2 (18.75%) and Level 4 (12.5%). The median MINORS score was 11.3 ± 2.2 in non-comparative studies and 17.7 ± 1.6 in comparative studies. These scores implied a reasonable level of evidence among the included studies. Two reviewers agreed with good reliability in the MINORS score (ICC = 0.974 [95% CI 0.834–0.992]).

**Table 1:** Characteristics of included studies

References	Country	Study design	Period	Diagnosis methods	Sample size (knees)	Age, y	BMI	Male (%)	Prevalence of OCIs (%)	Level of evidence	MINORS score
Beran et al. [40]	USA	Retrospective	NA	MRI	21 (22)	14.2	NA	76	72.7^e^	3	9
Fones et al. [41]	USA	Retrospective	2013.1–2018.12	MRI	118 (125)	13.9 ± 3.4	26.7 ± 6.9^a^ 25.0 ± 5.7^b^	52	57.6	3	17
Franzone et al. [17]	USA	Retrospective	2005.1–2010.3	Arthroscopy	38	21.0 ± 7.4	NA	34.2	63.2	3	10
Holliday et al. [42]	USA	Retrospective	NA	Surgery	231 (264)	24.2 ± 8.4	24.1 ± 4.1	29.4	84.5	3	10
Jiang et al. [18]	China	Retrospective	2016.1–2020.6	Arthroscopy	278	21.1 ± 4.6 (12–41)	NA	35.3	40.3	3	18
Jungesblut et al. [43]	Germany	Retrospective	2015.10–2020.3	Arthroscopy	141 (157)	5–17	21.6 ± 5.1	NA	59.2	3	10
Kolaczko et al. [44]	USA	Retrospective	2015–2020	MRI	61	16.13 ± 2.29^a^ 16.87 ± 8.65^b^	27.69 ± 9.74^a^ 26.41 ± 7.98^b^	NA	13.0	3	18
Palmowsk et al. [2]	Germany	Retrospective	2007.2–2012.9	MRI	50	23.2 ± 9.6 (11–50)	NA	66	84.0	3	11
Redler et al. [45]	USA	Prospective	2005–2015	MRI	171	22 (11–57)	NA	18.7	34.0	2	14
Stanitski et al. [46]	USA	Retrospective	NA	Arthroscopy	30	13.8 (12–16)	NA	43.3	56.7	3	10
Tompkins et al. [19]	USA	Prospective	2008–2012	MRI	157	23 ± 9.5^c^ 14.3 ± 1.4^d^	NA	50.3	83.5	4	14
Uimonen et al. [29]	Finland	Retrospective	NA	MRI	304	19.1 ± 7.3^a^ 21.0 ± 9.1^b^	NA	37.5	44.4	3	18
Zhang et al. [38]	China	Retrospective	2009.1–2012.6	US	49	24.5 (16–41)	NA	40.8	61.2^f^	3	10
Zhang et al. [47]	China	Prospective	2007.1–2015.6	MRI	140	14.3 ± 4.9 (9–17)	NA	42.1	46.8^ g^ 27.7^e^	2	15
Zhao et al. [48]	China	Retrospective	2011.1–2016.12	MRI	41	15.46 ± 1.86^a^ 15.31 ± 1.70^b^	NA	48.8	68.3	4	15
Zheng et al. [49]	China	Prospective	NA	MRI	115	9–41	NA	40.0	46.1^ g^ 27.0^e^	2	20

USA, the United States of America; NA, not available; MRI, magnetic resonance imaging; US, ultrasonography; MINORS, Methodological Index for Non-Randomized Studies

^a^Injuries group; ^b^Control group; ^c^Skeletally mature; ^d^Skeletally immature; ^e^Lateral femoral condyle; ^f^Inferomedial patella; ^g^Patella

### The association between demographic characteristics and OCIs

This study analyzed demographic risk factors for OCIs after patellar dislocation, including gender, skeletal maturation, age, and body mass index (BMI).

Seven studies reported the association between OCIs and gender [2, 17–19, 40, 43, 49]. Palmowsk et al. [2] found that male sex was a risk factor for OCIs (*p* = 0.029); however, two additional studies found no gender differences in patients with or without OCIs (*p* > 0.05) [17, 19]. In addition, Zheng et al. [49] found that patellar OCIs were more likely to be seen in males (*p* = 0.027), but no gender difference was found for OCIs in the lateral femoral condyle (LFC) (*p* = 0.123). Jungesblut et al. [43] identified male sex as an independent predictor associated with femoral OCIs (OR = 1.949, *p* = 0.022) but not patellar OCIs (*p* = 0.659). Beran et al. [40] also found male sex to be an independent risk factor for OCIs in LFC (OR = 3.609, *p* = 0.0174). Recently, a study by Jiang et al. [18] showed that male sex was an independent risk factor for OCIs (OR = 1.75, *p* = 0.028).

The association with OCIs was extracted from four studies for skeletal maturity [18, 19, 43, 49]. Tompkins et al. [19] concluded that skeletally mature individuals were more likely to develop OCIs (*p* < 0.05). Zheng et al. [49] found that a higher proportion of skeletal maturation in patients with patellar and femoral OCIs compared with those without lesions (*p* = 0.035 and *p* = 0.027, respectively), and skeletal maturation was determined to be an independent risk factor for patellar OCIs (OR = 2.324, *p* = 0.043). Jungesblut et al. [43] showed physeal closure as an independent predictor associated with the appearance of femoral OCIs (OR = 3.859, *p* = 0.042). However, Jiang et al. [18] showed that physeal closure was not associated with OCIs. Additionally, six studies [18, 41–45] reported the relationship between age and OCIs, and no studies found a significant association between them (*p* > 0.05), except for the study by Holliday et al. [42] (OR = 1.1, *p* = 0.001).

Four studies extracted the relationship between BMI and OCIs [41–44]. Holliday et al. [42] found that BMI was associated with OCIs by univariate logistic regression (OR = 1.1, *p* = 0.03). Jungesblut et al. [43] showed BMI ≥ 25 as an independent predictor associated with the appearance of femoral OCIs (OR = 1.406, *p* = 0.007). However, neither Kolaczko et al. [44] nor Fones et al. [41] found a difference in BMI between patients with and without OCIs (p = 0.49 and *p* = 0.456, respectively).

### The association between patellar depth and position and OCIs

Zhao et al. [48] extracted the correlation between the risk of OCIs and patellar depth and found no significant correlation (*p* = 0.593).

Eight studies reported the relationship between patellar position and OCIs [17, 18, 29, 41, 44, 45, 48, 49]. Both Redler et al. [45] (*p* = 0.303) and Zheng et al. [49] (*p* > 0.05) concluded that patellar height was not associated with OCIs, which was also consistent with the findings of Jiang et al. [18] (*p* = 0.390). Likewise, Franzone et al. [17] and Kolaczko et al. [44] did not find a correlation between patella alta and the presence of OCIs (*p* > 0.05 and *p* = 0.94, respectively). Regarding detailed measurement metrics (Table 2), all four studies [29, 41, 44, 48] found that CDI was not associated with OCIs (*p* > 0.05). Two studies [29, 48] showed that ISI was not associated with OCIs (*p* > 0.05). However, Uimonen et al. [29] showed a difference in PTI between patients with OCIs and those without OCIs (0.54 vs. 0.47, *p* < 0.001). Moreover, two studies [41, 48] showed no relationship between LPI and OCIs (*p* > 0.05). Notably, although Jiang et al. [18] found a statistically significant association between LPI and OCIs by univariate analysis (*p* = 0.014), there was no correlation in multiple logistic regression (OR = 0.91, *p* = 0.072). Zhao et al. [48] found that LPD was not associated with OCIs (*p* = 0.785). Two studies [44, 48] showed that LPFA and PFCA were not associated with OCIs (*p* > 0.05). More detailed information can be found in Table 3.

**Table 2:** The relationship between the OCIs and patellar height

References	CDI			ISI			PTI		
	OCIs	Control	*P*	OCIs	Control	*P*	OCIs	Control	*P*
Fones et al. [41]	1.3 ± 0.2	1.3 ± 0.2	0.199	NA			NA		
Kolaczko et al. [44]	1.19 ± 0.11	1.16 ± 0.17	0.68	NA			NA		
Uimonen et al. [29]	1.2 (1.16–1.23)	1.17 (1.13–1.20)	n.s	1.21 (1.17–1.24)	1.20 (1.17–1.23)	n.s	0.54	0.47	< 0.001
Zhao et al. [48]	1.30 ± 0.26	1.28 ± 0.23	0.861	1.26 ± 0.20	1.26 ± 0.15	0.944	NA		

*CDI* Caton–Deschamps index; *ISI* Insall–Salvati index; *PTI* Patellotrochlear index; *P P* Value; *n.s* Non-significant; *NA* Not available

**Table 3:** The correlation between the OCIs and other parameters of patellar position

References	LPI, °			LPD, mm			LPFA, °			PFCA		
	OCIs	Control	*P*	OCIs	Control	*P*	OCIs	Control	*P*	OCIs	Control	*P*
Fones et al. [41]	23.8 ± 9.0	21.2 ± 10.2	0.426	NA			NA			NA		
Jiang et al. [18]	24. 3 ± 3. 9	26. 8 ± 3. 2	0.014	NA			NA			NA		
Kolaczko et al. [44]	NA			NA			26.9 ± 11.9	22.6 ± 9.4	0.25	10.8 ± 4.6	12.0 ± 5.1	0.56
Zhao et al. [48]	17.81 ± 10.94	19.81 ± 9.19	0.544	6.89 ± 7.30	6.34 ± 5.35	0.785	14.68 ± 9.24	14.15 ± 6.84	0.837	24.34 ± 19.86	24.36 ± 16.59	0.998

*LPI* lateral patellar inclination; *LPD* lateral patellar displacement; *LPFA* lateral patellofemoral angle; *PFCA* patellofemoral congruence angle; *P P* Value; *NA* Not available

### The association between femoral trochlear morphology and OCIs

Eight studies reported the relationship between femoral trochlear morphology and OCIs [17, 29, 41, 42, 44, 45, 48, 49]. Fones et al. [41] showed that OCIs were associated with trochlear dysplasia defined by sulcus angle (OR = 1.06, *p* = 0.021). Holliday et al. [42] found that OCIs were related to both low-level trochlear dysplasia (OR = 2.9, *p* = 0.015) and high-level trochlear dysplasia (OR = 15.7, *p* < 0.001) by multiple logistic regression. However, Kolaczko et al. [44] found no difference regarding trochlear dysplasia in populations with or without OCIs (*p* = 0.72). Likewise, both Redler et al. [45] and Franzone et al. [17] also found that trochlear dysplasia was not associated with OCIs (*p* = 0.843 and *p* > 0.05, respectively). Interestingly, Zheng et al. [49] found that normal femoral trochlea was an independent risk factor for OCIs of the patella (OR = 3.835; *p* = 0.01) and LFC (OR = 3.347; *p* = 0.029), in contrast to all of the above findings. Similarly, Uimonen et al. [29] also concluded that trochlear configuration assessed by TFAR (*p* < 0.001) and TCAR (*p* = 0.013) was closer to normal in patients with OCIs than those without OCIs.

Further, to explore whether a single quantitative imaging metric of trochlear morphology would be predictive, this study was summarized separately (Table 4). For trochlear depth, Uimonen et al. [29] found a shallower trochlear depth in the OCIs group than without OCIs (2.5 vs. 3.0, *p* < 0.001), while Zhao et al. [48] concluded that it was not statistically different between the two groups (*p* = 0.616). Among the four studies [29, 41, 44, 48] on sulcus angle and OCIs, only Fones et al. [41] identified a significant association between increased sulcus angle and the incidence of OCIs (159.8 ± 9.1 in the OCIs group vs. 155.3 ± 8.3 in the control group, *p* = 0.021), with the remaining studies finding no statistical difference regarding sulcus angle for group comparison (*p* > 0.05). All three studies [29, 41, 48] showed no correlation between LTIA and OCIs (*p* > 0.05). In addition, Uimonen et al. [29] also demonstrated a statistically significant difference in both TFAR (0.54 vs. 0.43, *p* < 0.001) and TCAR (1.04 vs. 1.05, *p* = 0.013) between the OCIs group and the control group.

**Table 4:** Quantitative imaging metrics related to trochlear morphology

References	Trochlear depth, mm			Sulcus angle, °			LTIA, °			TFAR			TCAR		
	OCIs	Control	*P*	OCIs	Control	*P*	OCIs	Control	*P*	OCIs	Control	*P*	OCIs	Control	*P*
Fones et al. [41]	NA			159.8 ± 9.1	155.3 ± 8.3	0.021	2.3 ± 9.7	5.8 ± 12.3	0.546	NA			NA		
Kolaczko et al. [44]	NA			126.34 ± 12.9	128.13 ± 11.1	0.68	NA			NA			NA		
Uimonen et al. [29]	2.5	3	< 0.001	154.5	155.1	n.s	13.5	14.3	n.s	0.54	0.43	< 0.001	1.04	1.05	0.013
Zhao et al. [48]	9.54 ± 2.66	10.08 ± 3.40	0.616	148.81 ± 6.23	150.72 ± 10.18	0.464	15.15 ± 4.82	12.59 ± 4.78	0.119	NA			NA		

*LTIA* Lateral trochlear inclination angle; *TFAR* Trochlear facet asymmetry ratio; *TCAR* Trochlear condyle asymmetry ratio; *P P* Value; *n.s* Non-significant; *NA* Not available

### Summary of additional risk factors

For tibial tubercle–trochlear groove (TT-TG) distance, five studies [18, 29, 41, 45, 49] have reported an association with the risk of OCIs. Zheng et al. [49] showed that normal TT-TG distance was an independent risk factor for patellar OCIs (OR = 2.824; *p* = 0.031). However, Fones et al. [41] argued that there was no association between OCIs and distal TT-TG distance (*p* = 0.600) or proximal TT-TG distance (*p* = 0.556). Redler et al. [45] and Jiang et al. [18] concluded that TT-TG was unrelated to OCIs (*p* = 0.874 and *p* = 0.292, respectively). Likewise, Uimonen et al. [29] also revealed no difference in TT-TG between patients with OCIs and those without OCIs (*p* > 0.05).

Concerning ligamentous laxity or articular hypermobility, five studies [17, 42, 44–46] have reported its association with the risk of OCIs. Redler et al. [45] concluded that although the percentage of OCIs did not differ between patients with and without ligamentous laxity (*p* > 0.05), patients with ligamentous laxity rarely had severe OCIs (grade 3 or 4) of the patella (45% vs. 74%, *p* = 0.004) or femur (13% vs. 67%, *p* = 0.05) compared to those with no laxity. Stanitski et al. [46] found that articular hypermobility decreased the risk of OCIs by approximately 2.5 times (33% vs. 80%) compared to the control group after acute patellar dislocations. Similarly, Holliday et al. [42] found a Beighton score ≥ 4 (indicating articular hypermobility) showed a protective effect on the patellofemoral joint in a univariable logistic regression (OR = 0.36; *p* = 0.009). However, Neither Kolaczko et al. [44] nor Franzone et al. [17] found a correlation between ligamentous laxity and the presence of OCIs (*p* = 0.49 and *p* > 0.05, respectively).

Regarding factors such as the duration of symptoms or the number of dislocations, three studies [17, 41, 42] have reported its association with the risk of OCIs. Franzone et al. [17] suggested that although chronicity of patellar instability greater than five years was associated with trochlear lesions (*p* < 0.05), a multivariate regression analysis subsequently demonstrated that the chronicity of patellar instability did not predict OCIs of the patella and trochlea. Fones et al. [41] also concluded that the symptom duration of any instability after the initial dislocation was not statistically significant with the presence of OCIs (127.1 in the OCIs group vs. 268.1 in the control group, *p* = 0.113). Likewise, Holliday et al. [42] found no significant association between the number of dislocations and the presence of OCIs (*p* = 0.99).

In addition, two studies [38, 47] on medial patellofemoral ligament (MPFL) injury patterns and OCIs. In 2013, Zhang et al. [38] found a significant difference between partial and complete MPFL injuries in the incidence of patellar OCIs (*p* = 0.035). Subsequently, Zhang et al. [47] further found that complete MPFL tears predispose to a higher grade of patellar OCIs than partial MPFL injuries (*p* < 0.05).

Moreover, Jiang et al. [18] found that increased femoral anteversion angle was an independent risk factor for patellar dislocation combined with OCIs (OR = 3.12, *p* = 0.012). Jungesblut et al. [43] also suggested that traumatic mechanisms lead to more patellar (OR = 7.083, *p* = 0.033) and femoral OCIs (OR = 42.17, *p* < 0.001). Kolaczko et al. [44] also found that effusions were the factor that showed a statistically significant association with occult OCIs (*p* = 0.02).

## Discussion

This systematic review found that an increased risk of OCIs following patellar dislocation may be associated with male sex and skeletal maturation. Furthermore, normal femoral trochlea, complete MPFL injuries may increase the risk of OCIs, while factors such as ligamentous laxity or joint hypermobility may reduce the risk.

In acute patellar dislocation, the shearing mechanism can lead to patellofemoral joint injury. When the patella is relocated, the articular surface of the patellofemoral is at risk for further damage due to the convex patellar articular surface and the concave trochlear groove [38, 50]. A comprehensive understanding of patellofemoral joint morphology and patellofemoral motion allowed us to better study risk factors of OCIs after patellar dislocations.

Regarding demographic characteristics, the systematic review showed that the increased risk of OCIs was likely associated with male sex and skeletal maturity but not with patient age. Besides, it remained unclear whether a greater BMI increases the risk of OCIs based on the available evidence, and further studies are needed in the future. Gender makes a difference in patellar dislocation, and female patients are one of the risk factors for patellar dislocation [51]. However, in patients with patellar dislocations combined with OCIs, the study found that the risk seemed higher in males, possibly because female patients tend to have higher joint laxity, allowing for less impingement of the patellofemoral joint during the dislocation. In contrast, male patients experience higher shear stresses during dislocation and repositioning, which are more likely to cause OCIs [52]. The anatomy of the patellofemoral joint is not fully developed and perfect in skeletally immature patients, and the MPFL and internal femoral oblique muscles are not fully fused [53–55]. Thus, patellofemoral joint instability in skeletally immature patients means fewer forces are needed for dislocation and fewer OCIs than in skeletally mature patients.

Many studies reported that abnormal anatomy of the patella and femoral trochlea is an independent risk factor for patellar dislocation [56–58]. Accordingly, pathoanatomical parameters of a patellofemoral joint may theoretically exacerbate the tendency for patellar dislocations, but whether they would also aggravate OCIs after patellar dislocation was still unclear. The study can establish an association between femoral trochlear morphology and OCIs by integrating existing evidence. In more detail, Fones et al. [41] showed that OCIs were closely associated with trochlear dysplasia via the sulcus angle. Holliday et al. [42] showed that trochlear dysplasia was the leading risk factor for OCIs in patients with patellar dislocation. However, opposing viewpoints also abound. On the one hand, both Redler et al. [45] and Franzone et al. [17] concluded that trochlear dysplasia was not associated with the occurrence of OCIs, which findings were also in line with the study of Kolaczko et al. [44]. On the other hand, Zheng et al. [49] yielded the novel finding that normal femoral trochlea was a risk factor for OCIs after patellar dislocations. Further, since people with normal femoral glides have good patellofemoral joint stability, if they experience patellar dislocation, their outward traction and retraction forces are stronger and more likely to lead to OCIs [49, 59]. Similarly, Uimonen et al. [29] also concluded that patients with OCIs had a trochlear configuration closer to normal anatomy than patients without OCIs. Therefore, the authors tended to support that normal femoral trochlea was more likely to lead to OCIs in patients with patellar dislocation. Most evidence indicated that femoral morphologic correlates (trochlear depth, sulcus angle, and LTIA) did not differ significantly in patients with or without OCIs.

Furthermore, the study concluded that no relationship existed between the risk of OCIs and patellar depth and position. The above findings may also be related to the insufficient number of included studies and the measurement accuracy of the relevant indicators. The patella is capable of varying degrees of displacement in many positions, and the methods used to determine patellar position are not always precise, so inevitable measurement errors end up occurring during imaging evaluation [31, 60]. Therefore, we expect further anatomical studies to create a new measurement method. Likewise, we also hope that future original studies could expand the sample size along with improving the accuracy of the measurement of imaging metrics to resolve the problem more objectively.

Additionally, the force required to develop patellar dislocation may be lower in patients with longer TT-TG than those with shorter TT-TG. As a result, OCIs were more common theoretically in patients with normal TT-TG [29]. Zheng et al. [49] indeed concluded that patients with a normal TT-TG distance were more likely to develop patellar OCIs. However, plenty of research concluded that TT-TG was also unrelated to OCIs [18, 29, 41, 45]. Consequently, the authors preferred that TT-TG was not a risk factor in OCIs after patellar dislocations. Interestingly, after the above analysis and summary, we were surprised to find that those considered critical anatomical risk factors for patellar dislocation (patella alta, trochlear dysplasia, and elevated TT-TG distance) were not risk factors for OCIs after patellar dislocation [58].

Regarding factors such as ligamentous laxity or joint hypermobility, the study concluded that they could reduce the risk of OCIs based on the available evidence. Since the final result of ligamentous laxity or articular hypermobility was articular instability, less force is required for acute dislocation. Therefore, the reason for their ability to reduce the risk of OCIs is not difficult to understand, in line with the previous points made by the authors.

Interestingly, repeated dislocations theoretically result in abnormal contact pressures in the patellofemoral joint, which leads to increased wear of the articular surface and an increased incidence of OCIs [37, 42, 61]. However, this study suggested that the duration of symptoms or the number of dislocations did not increase the risk of OCIs based on the existing studies. The authors speculated that the incidence of OCIs after the first patellar dislocation is inherently high [2, 62], resulting in no significant clinical correlation between increased chronicity of instability or the number of dislocations and increased risk of OCIs.

For the MPFL injuries pattern, we tended to consider that complete MPFL injuries predisposed to more severe OCIs than partial MPFL injuries. The strength of the force was critical in determining the severity of the articular injury [47]. The more severe the injury to the MPFL meant the more powerful the shift force and distraction force applied to the MPFL, and the greater impingement force applied to the patellofemoral joint surface, the more likely it was to result in OCIs [38]. Accordingly, careful evaluation of the patellofemoral joint with complete MPFL injuries is vital, as OCIs may occur more commonly in this case than no or partial MPFL injuries.

Moreover, due to the limited number of included studies, the authors could only tentatively conclude that increased femoral anteversion, traumatic mechanisms, and effusions had predictive roles for the appearance of OCIs after patellar dislocations. However, more studies are still needed to verify this in the future. Recently, other parameters associated with OCIs in non-patellar dislocations were explored, including the trochlear sulcus depth [63], the angle of Fulkerson [63], and femoral antetorsion [64]. Nevertheless, the applicability of the above metrics to patellar dislocations remains to be determined. Besides, an increasing number of predictors of recurrent patellar dislocation, including wiberg index [65] and patellar shift ratio [66], have emerged as research progresses. Whether they can further predict OCIs after patellar dislocation holds excellent clinical significance and promise.

The findings of this systematic review have promising clinical implications. Orthopedic surgeons must be aware of male and skeletally mature patients with patellar dislocation, as these populations are more likely to be accompanied by OCIs. Moreover, knowledge of anatomically relevant risk factors has crucial diagnostic value for OCIs after patellar dislocations. For example, normal femoral trochlea and complete MPFL injuries may increase the risk of OCIs. In contrast, factors such as ligamentous laxity or joint hypermobility may decrease the risk. All in all, understanding the risk factors associated with OCIs after patellar dislocations can contribute to more accurate preoperative diagnosis and better surgical planning and help clinicians guide patient counseling regarding long-term prognosis.

## Limitations

This study is not without limitations. First, perhaps the risk factors of OCIs differ between primary and recurrent patellar dislocations; however, this study did not categorize them. Therefore, subsequent studies need to explore risk factors and preventive measures according to the pathogenesis of different dislocation-related OCIs. Second, the reported risk factors associated with OCIs after patellar dislocation were diverse. However, some of these factors have been relatively poorly studied, making it difficult to draw definitive conclusions about them. Third, this systematic review followed the PRISMA guidelines, but due to the heterogeneity of the study design and extracted information, a quantitative meta-analysis was not performed.

## Conclusions

Based on the available evidence, an increased risk of OCIs following patellar dislocation may be associated with male sex and skeletal maturation. Furthermore, normal femoral trochlea, and complete MPFL injuries may increase the risk of OCIs, while factors such as ligamentous laxity or joint hypermobility may reduce the risk.

## Data Availability

All data generated or analyzed during this study are included in this published article.

## Abbreviations

OCIs: Osteochondral injuries
CNKI: China national knowledge infrastructure
PRISMA: Preferred reporting items for systematic reviews and meta-analyses
BMI: Body mass index
TT-TG: Tibial tubercle–trochlear groove
MPFL: Medial patellofemoral ligament
MeSH: Medical subject headings
MINORS: Methodological index for non-randomized studies
ICC: Intra-class correlation coefficient
CDI: Caton-deschamps index
ISI: Insall-salvati index
PTI: Patellotrochlear index
LPI: Lateral patellar inclination
LPD: Lateral patellar displacement
LPFA: Lateral patellofemoral angle
PFCA: Patellofemoral congruence angle
LTIA: Lateral trochlear inclination angle
TFAR: Trochlear facet asymmetry ratio
TCAR: Trochlear condyle asymmetry ratio
LFC: Lateral femoral condyle
